# High‐frequency (20 MHz) high‐intensity focused ultrasound: New ablative method for color‐independent tattoo removal in 1‐3 sessions. An open‐label exploratory study

**DOI:** 10.1111/srt.12885

**Published:** 2020-06-18

**Authors:** Jørgen Serup, Torsten Bove, Tomasz Zawada, Alexander Jessen, Mattia Poli

**Affiliations:** ^1^ Department of Dermatology Bispebjerg University Hospital Copenhagen Denmark; ^2^ TOOsonix A/S Horsholm Denmark

**Keywords:** adverse event, effectiveness, guidance, pain, picosecond laser, procedure, scar, tattoo pigment, wound healing, YAG laser

## Abstract

**Background:**

High‐intensity focused ultrasound (HIFU) operating at 20 MHz is new and potentially applicable to ablative tattoo removal. The method was documented safe and rational in preclinical testing.

**Materials and Methods:**

High‐intensity focused ultrasound was introduced to subjects when lasers and dermatome shaving had failed or caused side effects. Transducers with focal depths between 1.1 mm and 1.7 mm in the skin were used, and settings of 0.4‐1.2 J/shot at pulse durations of 150 ms were applied. Tattoos were covered with synergistic “shoulder‐by‐shoulder” focused ultrasound shots. Effectiveness and side effects were measured.

**Results:**

Twenty‐two subjects with 67 tattoos were treated. 62% benefitted (19% cleared, 43% partially cleared), and 28% had minor effect. VAS pain was 5‐6 versus 7‐9 with previous lasers removal. Wound healing was longer after HIFU ablation (1‐3 months). 57% of subjects had no scar or minor visible changes of skin surface markings only, while 19% had moderate or major skin thickening. Hypertrophic scar or keloid scars were not observed.

**Discussion/Conclusion:**

High‐intensity focused ultrasound was effective in removal of difficult tattoos of any color where Nd:YAG lasers had failed. The method only needs 1‐3 sessions. As an ablative method, the wound healing period is longer than with laser removal and needs attention. Focused ultrasound can be used as a first‐line treatment of smaller tattoos independent of color, and second line when Nd:YAG lasers have failed or caused problems. The operator shall be qualified, as with lasers.

## 1 INTRODUCTION

The current trend for people having one or more tattoo is unfortunately paralleled by an increasing need for tattoo removal due to regret, tattoos‐associated disease, or for social reasons.^1^, ^2^ Tattoo removal has a long history. In the past abrasion by rubbing with salt (salabrasion), exposure to acids and caustic chemicals, and burning the tattooed skin with cigarettes were practiced by the those who regretted. Medical practitioners used surgical excision as wells as dermabrasion and salabrasion, however with limitations due to size, anatomical site and sequelae observed particularly as scars.

Today, nanosecond Q‐switched lasers, for example, Nd:YAG, alexandrite, and ruby lasers, are used as the methods of choice, recently followed by the launch of picosecond lasers.^3^, ^4^, ^5^ Lasers are of different wavelength and therefore have color‐ and pigment‐dependent energy absorption, which is an advantage and a limitation at the same time. Some colors, particularly yellow, green, and blue, have low energy absorption to available laser wavelengths and are therefore difficult to remove. White titanium pigment used as pure pigment or in toned ink may turn dark after high‐energy laser exposure. Lasers may furthermore produce photochemical effects on the tattoo pigment with formation of breakdown substances that are allergenic, carcinogenic, or otherwise toxic.^6^, ^7^ Lasers produce a focused ultra‐short thermal burn with temperatures reaching 300‐400°C in the pigment. These high temperatures are conducted to nearby skin structures, which are then damaged. For this reason, laser treatment often is very painful, and the thermal insult is followed by a wheal and flare reaction. The long‐term sequelae are consequently scarring and dyspigmentation with hypo‐ or hypermelanosis.^8^

Lasers are, despite these known limitations, positioned as the gold standard treatment of today. Ablative CO_2_ lasers are used only exceptionally due to the high risk of scars. Lasers shall not be applied to allergic tattoo reactions, since the reaction can be boosted through neoformation of allergenic breakdown products.^9^ Allergic reactions are better treated with dermatome shaving.^10^ Caustic, strongly acidic, or strongly oxidant products for tattoo removal are marketed as cosmetic products, and on free sale despite the obscure dose‐effect control and the very high risk of scars.^11^, ^12^ Laser removal requiring 8‐12 treatment sessions, and given with a minimum interval of 6 weeks, is expensive and cumbersome to fulfill, and there is therefore a need for new or supplementary methods.

The new method for tattoo removal presented in this article is innovative and based on high‐intensity focused ultrasound (HIFU). HIFU systems operating at frequencies from 500 kHz to approximately 3 MHz were since decennia established for non‐invasive treatment of a variety of medical indications exemplified by internal cancers of major organs and cerebral pathologies, with the HIFU focal point located deep within the body or the brain.^13^, ^14^, ^15^, ^16^, ^17^, ^18^, ^19^, ^20^, ^21^ Selective HIFU treatment of the thalamus has revolutionized the treatment of invalidating essential tremor. HIFU used at even lower frequencies can be used to treat kidney stones. In all such treatments, accurate positioning of the focal point at targets within organs is guided by magnetic resonance imaging or ultrasound imaging. Thus, the principle of targeted and surveyed treatment using HIFU is verified and internationally accepted.

The size of the focal zone generated by a HIFU transducer is inversely dependent on the operating frequency, that is, the higher the frequency the smaller the focal zone. For HIFU treatments to be relevant within the field of dermatology, the focal zone must be small to match the 1‐2 mm total thickness of the human skin. This requires an operating frequency of at least 15 MHz as confirmed by theoretical considerations and preclinical testing.^22^, ^23^

Commercially available HIFU systems for aesthetic wrinkle reduction and body contouring operate at about 3‐10 MHz. Such frequencies create focal zones that exceed the total thickness of the skin, and are therefore not feasible for dermatology indications addressing the dermal end epidermal layers only.^24^, ^25^

The ultrasonic device introduced for tattoo removal in this article is based on 20 MHz high‐intensity focused ultrasound (HIFU).^26^ The HIFU device uses a focused acoustic transducer with a concave surface that concentrates ultrasound beams over pulse durations of typically 100‐200 ms in a very narrow focal zone. In the focal zone, where the convergent beam concentrates, the energy density is greatly intensified with a resultant rapid heating of the tissue temperature up to approximately 65°C, which induces a highly localized acute necrosis. It shall be emphasized that the dept control of the thermal lesion in a medium is optimized; that is, the delineation in depth is reproducible and sharp under a given setting of the instrument.

**Figure 1:**
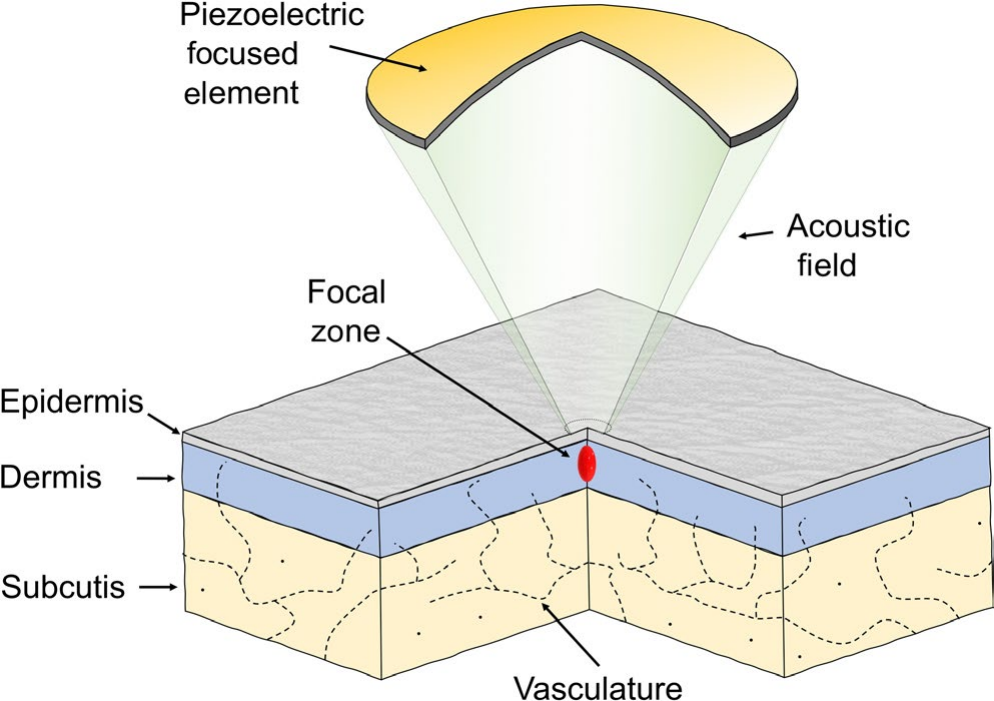
Schematic illustration of the high‐intensity focused ultrasound (HIFU) principle. The transducer is a focused piezoelectric element constructed to deliver non‐invasive acoustic energy of high intensity into a confined focal zone under the skin surface. The zone of concentrated heating to about 65°C is in the focal point, and the adjacent part of the medium above the focal point. The energy attenuates abruptly below the focal point. Exposed to 65°C, cells die and the vitality of the exposed skin is lost

The method is “color blind” and “content‐neutral,” as the thermal lesion has no special preference in the compartment or layer of the skin exposed to HIFU treatment. Different transducers can be selected to accurately position the lesion as preferred at any vertical depth of the skin. Thus, the method can be adapted for both individual differences in skin thickness and the vertical position of a lesion. With the focal point positioned in the outer skin, the method is ablative. With the focal point in the deep dermis, at the interface to subcutis or directly in the subcutis the method is non‐invasive and non‐ablative, see Figure 1.

Laboratory study and preclinical study in Göttingen pigs have demonstrated reproducibility and a linear energy‐dependent dose‐effect curve. Preclinical testing indicated a realistic range of operation within a dose range of 0.6‐1.5 J/dose in the clinic.^23^, ^26^ The method produced the intended thermic insults in the dermis and was confirmed as ablative or non‐ablative depending on setting and choice of transducer. In the pig model, scarring was not a problem, albeit histology revealed subclinical fibrous change in cases exposed to high‐energy multiple doses positioned in close proximity to each other.

Such horizontal synergy between ultrasound lesions applied nearby each other, that is, *“shoulder by shoulder”* mode, was compared to *single dose,* the latter clearly having lower effect.^23^, ^27^ Thus, the dose delivered to the skin, as determined by instrument setting, had an additional operator‐dependent dimension, namely the number and closeness of applied treatment doses.

This horizontal synergy is important in the practical use of the method for clinical applications. Synergy can also be produced in the vertical plane by *“double pass”* mode of use, that is, administering double dosing over the same site within a minimum time interval, thereby deliberately increasing the tissue temperature and affected volume. A third option is use of two different probes with different depths of the focal point on the same site, thus creating a *“sandwich”* mode of use.

The 20 MHz HIFU system used in the present study has recently been successfully introduced to actinic keratosis and selected cases of basal cell carcinoma and Kaposi sarcoma.^28^

**Figure 2:**
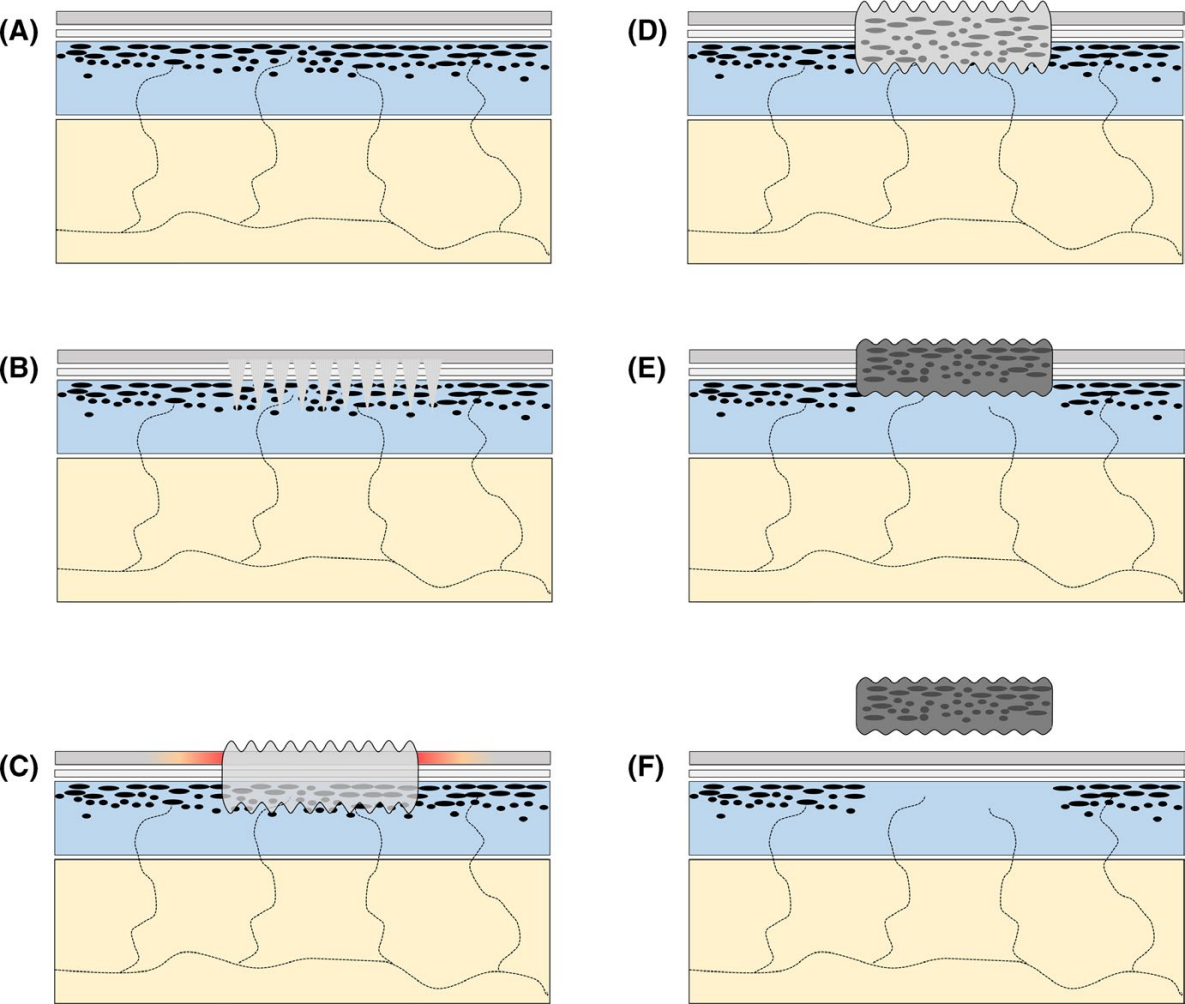
Schematic illustration of ablative effects of 20 MHz high‐intensity focused ultrasound (HIFU) treatment dosed “shoulder by shoulder” according to preclinical study of pigs, extrapolated to treatment of tattoos.^27^ The illustration shows the hypothetical mode of action and sequence of events of tattoo removal using 20 MHz HIFU. A, cross‐sectional illustration of the skin with black tattoo pigment concentrated in the outer dermis, which is a typical location. B, HIFU dosed “shoulder by shoulder” and positioned to target the outer dermis. C, immediate wheal and flare reaction due to the thermal ultrasound trauma causing histamine release; edematous center and perilesional axonal flare. D, HIFU‐induced thermal necrosis of the outer dermis, holding the pigment in the necrotic mass. E, scab and loosened necrotic material. F, the necrotic material, with tattoo pigment, is expelled from the skin leaving an excavated inflamed wound that will heal gradually. Inflammation of the wound and the border of the wound are not illustrated. While the wound is open, with broken barrier and secretion, tattoo pigment particles still can migrate out of the skin into the bandage. Thus, clearing of pigment has two phases, that is, a first via the necrotic crust and later via the post‐necrotic open wound until the epidermis and the barrier are restored, and an additional mode of metabolic breakdown of pigment affected by inflammation of the wound bed extending into the border of the wound

The HIFU treatment, as found in the study of pigs, was often followed by a wheal and flare response that peaked after 5‐10 minutes and faded over the subsequent 10‐30 minutes. After a few days a superficial dry wound and inflammatory reaction in the treated area was observed, followed by the necrotic material being expelled from the skin leaving an excavated inflamed wound that healed gradually. Based on these observations from the animal study, a hypothetical mode of action of HIFU applied to tattoo removal was deducted according to the process shown in Figure 2.

The study presented below is the very first study of the novel 20 MHz HIFU method applied to tattoo removal according to this mode of action. A clinical material of challenging cases of tattoo removal that mostly had been unsuccessfully treated with lasers before is presented including illustrative case reports. Thus, this open‐label exploratory study primarily addressed difficult‐to‐remove tattoos.

## 2 MATERIAL AND METHODS

### 2.1 Patient recruitment

The study was open‐label and integrated in the ongoing clinical treatment practiced at the Department of Dermatology of Bispebjerg University Hospital, Denmark. The department has a specialized Tattoo Clinic treating tattoo complications, and a laser section which on special indications performs Nd:YAG‐laser removal of tattoos.^29^, ^30^, ^31^, ^32^ The surgical unit has special experience in removal of tattoos with allergic reactions to red azo pigment by dermatome shaving.^10^ HIFU treatment was offered as an optional method of tattoo removal to patients with problems and failures after previous removal by other methods, for example, when lasers were ineffective, when the color of the tattoo did not match the wavelength of the laser, when scarring was limiting laser efficacy, when pain associated with lasers was unbearable, when there was an urgent need of treatment in a few sessions (gang members under resocialization threatened to have special symbols removed), and on other special indications. Thus, fixed standards of in‐ and exclusion criteria were not used. The material was consecutive and included every treated patient. The study was an open‐label production control assessment. The study was conducted from November 2018 to February 2020. Patients were informed before treatments and gave their consent. The principles of the Helsinki Declaration II were followed.

### 2.2 Equipment

Treatments of this study were performed using the novel System ONE from TOOsonix A/S, Denmark.^22^, ^26^

The system operates at 20 MHz ± 5%. The HIFU equipment was safety tested and approved and registered by the Dept. of Medical Engineering of the Hospital. The equipment fulfills the general requirements for basic safety and essential performance according to IEC 60601‐1:2006 including its collateral standards.

**Figure 3:**
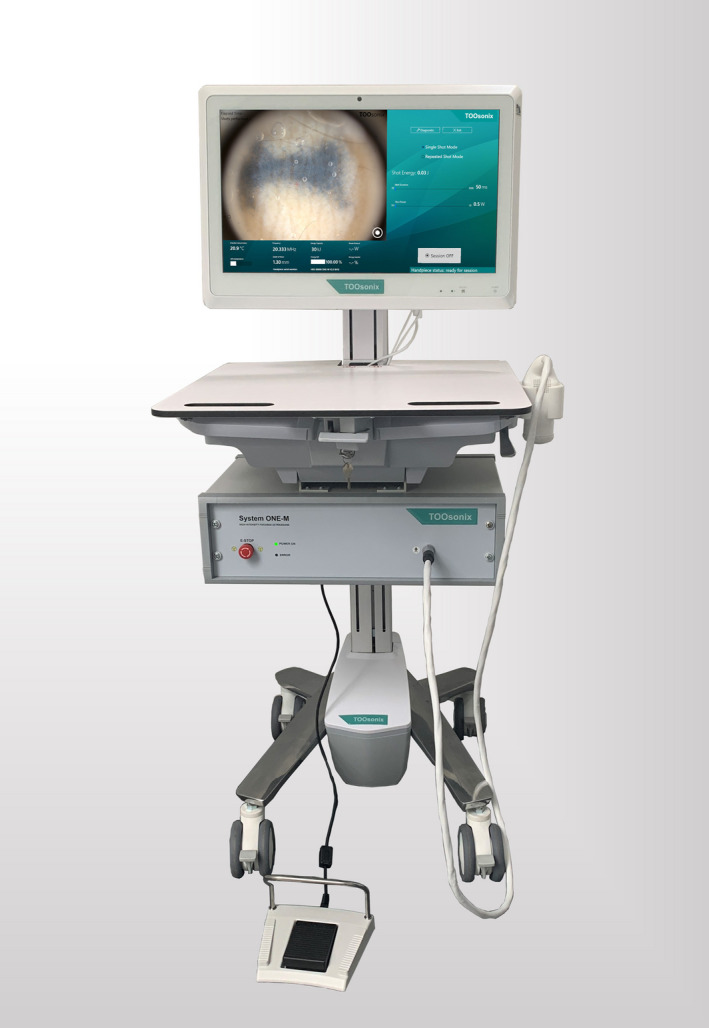
TOOsonix 20 MHz high‐intensity focused ultrasound (HIFU) System ONE used in the study for tattoo removal. A range of transducers and probes are available

The system, shown in Figure 3, consists of an ultrasound power unit responsible for generation and regulation of ultrasound signals, handpieces with a range of ultrasound transducers, and a software to manage treatment settings. HIFU doses, or “shots,” are activated manually by a footswitch. High‐resolution real‐time monitoring of the treated area is integrated in the system using a digital video camera that operates as a dermoscope. Different handpieces of the system are characterized by their −6 dB focal zone depth, that is, the maximum extend of the zone where acoustic intensity is within 25% of the maximum intensity in the center of the zone. Handpieces with focal depths ranging from 1.1 to 2.7 mm were available and selected depending on the tattoo under treatment. The treatment for tattoo removal was primarily intended to be ablative, and handpieces were therefore chosen accordingly.

### 2.3 Procedure of tattoo removal

Prior to treatment, the transducer chamber was filled with non‐gaseous distilled water and closed with a thin polyethylene film. A standard ultrasound coupling gel was used between the skin surface and the probe.

The duration of each ultrasound shot was chosen at 150 ms in all treatments. This has previously been found adequate for sufficient energy transfer, and at the same time minimize influence of movement of the handpiece during HIFU transmission. The acoustic peak energy was preferably 0.6, 0.9 or 1.2 J/shot depending on the thickness of the tattooed skin and the anatomical site.

**Figure 4:**
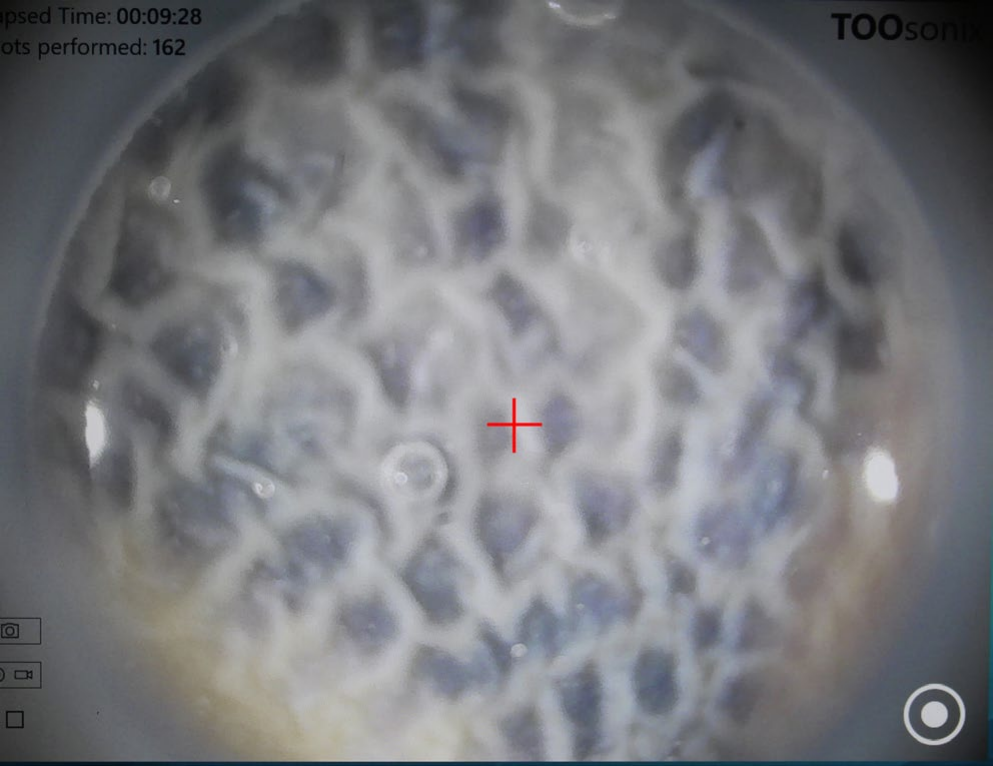
Close‐up screen photograph of the dermoscopy picture of a black tattoo treated with “shoulder by shoulder” shots. The red cross at the center is the marker of position being treated when an ultrasound shot is fired

Guided by a red pointer on the screen and observing the skin surface with the integrated dermoscope camera, the HIFU shot was positioned precisely over the target in the tattoo. The system was activated with the footswitch, and a shot was fired. Whitening or contraction of the treated skin was displayed directly on the screen in real‐time and surveyed to control that an effective dose was taken up by the tattoo (Figure 4).

A full treatment consisted of consecutive shots administered shoulder‐by‐shoulder, that is, placed with approximately 1‐2 mm between their centers, to fully cover the targeted area. Shots were administered at intervals of approximately 1‐2 seconds. Optimal dosing requires the probe to be held precisely perpendicular to the skin surface. A boiling sound as a shot is fired indicates the angle was not right, and air bobbles in the coupling gel had absorbed the energy.

Treatments included 1‐2 shots in the surrounding skin immediately outside the tattoo to enforce treatment of the demarcation of the tattoo, for example, the “lining.” The tattoo contour or lining, most often made in black, is normally made by the tattooist with a thin liner needle that installs the pigment rather deep in the dermis. Thin lines in tattoos, such as texts, were deliberately treated in the surrounding skin as well to benefit from the synergy of “shoulder by shoulder” applications.

### 2.4 Clinical assessment and rating scales

Immediate *wheal and flare reaction* directly after treatment was rated as follows: 0 no reaction, 1 + redness only, 2 + redness and edema, 3 + perilesional redness in the surrounding skin additional to redness and edema. 1 + and 2 + are histamine mediated; the flare of 3 + is an axonal reflex not accompanied by edema.

High‐intensity focused ultrasound treatment causes *instant pain* directly when a shot is fired. Pain was measured on a visual analogue scale, ranging from 0 to 10 with 10 marking unbearable pain.

The *effectiveness of HIFU on tattoo color reduction* was rated as follows: “cleared” (no remnant pigment), “partly cleared” (removal or diminishment of up to half of the pigment or the color of the tattoo), “minor effect” (removal or diminishment of less than half of the pigment or the color of the tattoo), and “no effect” meaning no visual effect on color or pigment.

*Scar at follow‐up* was rated as follows: no scar, 1 + visible change of skin surface markings only, 2 + slightly increased skin thickness with slightly increased skin tension, 3 + moderate skin thickening with definitely increased tension, 4 + major thickening and tight skin, 5 + hypertrophic scar or keloid. Rating of scar was not conducted at start of study, and outcome rating of scar is thus the accumulated sum of scarring resulting from tattoo needle trauma, previous laser or dermatome shaving and, finally, HIFU used as rescue intervention.

## 3 RESULTS

The study included 22 subjects with 67 tattoos treated with 20 MHz HIFU. Most tattoos were black and made in a tattoo parlor, but also red and green tattoos had been included. One had gun powder tattoos in the face, and one subject had X‐ray field markings made by a hospital.

Most tattoos were recalcitrant and had been treated before with Q‐switched Nd‐YAG lasers or dermatome shaving, the latter applied as first‐line treatment of red tattoos with allergic reactions.

Subjects in the study were treated with 1‐3 HIFU sessions with an interval of no less than 6 weeks to observe the immediate outcome. While different probes were used for special cases, the preferred probes had focal depths of 1.1 and 1.3 mm to obtain an ablative treatment mode. The preferred power settings were 0.9 and 1.2 J/shot. Follow‐up ranged from 3 to 12 months.

The results of the study are summarized in Table 1, and clinical findings are discussed below. The process of tattoo removal by HIFU is furthermore exemplified in selected case reports.

**Table 1:** Overview of results in 22 subjects with 67 tattoos treated with high‐intensity focused ultrasound aiming at tattoo pigment removal

Subject	Diagnosis	No of Lesions (Pre‐treatment)	Probe/Energy (Joule)	VAS Pain Median, (Range)	Outcome
DA	Black tattoos	2 (Laser tried)	1.3 mm/ 1.2 J	5	Cleared, No scar
AS	Black tattoos	2 (Laser tried)	1.3 mm/ 0.9‐1.2 J	0.5 (EMLA^®^)	Remnant pigment, 1 + scar
JK	Gun powder	4 (Laser tried)	1.3 mm/ 0.9‐1.2 J	3.5 (3‐4)	No effect No sequelae
CG	Black tattoos	8 (Laser tried)	1.7 mm/ 0.9‐1.5 J	4 (3‐6)	Cleared, some sites with remnant pigment, No scar
MJ	Red Allergic	3, 3 (Shaving)	1.7 mm/0.4‐0.9 J 1.7 mm/1.2 J	2 (1‐3)	Remnant pigment, 2 + scar
BH	Black tattoo	1 (Untreated)	1.3 mm/ 0.9 J	6	Minor effect, 2 + scar
PH	Black tattoo	1 (Untreated)	1.3 mm/ 0.9 J	3.5 (3‐4)	Minor effect, 2 + scar
OW	Green tattoo	1 (Salabrasion and Laser tried)	1.3mm/ 1.2 J	2.5 (2‐3)	Partly cleared, 1 + scar
SK	Black/colored tattoos	3 (Laser tried)	1.3 mm/ 0.9‐1.2 J	3 (1‐7)	Partly cleared, 1 + scar
CC	Black/colored tattoos	4, 1 (Shaving)	1.3, 1.7 mm/ 1.2 J	3 (1‐6)	Minor effect, 2 + scar
MB	Black tattoos	7 (Untreated)	1.3 mm/ 0.9‐1.5 J	3 (2‐7)	Partly cleared, 1 + scar
TS	Red/black tattoo	1 (Laser tried)	1.3 mm/ 0.9‐1.5 J	1.5 (1‐2)	Partly cleared No scar
TJ	Black marks Radiotherapy	3 (Untreated)	1.3 mm/ 1.2 J	0.5	Cleared No scar
PA	Red/brown/green tattoos	5 (Laser tried)	1.3 mm/ 0.4‐0.6 J	1‐2	Partly cleared No scar
SP	Black tattoo	1 (Laser tried)	1.3 mm/ 1.2 J	2 (0‐4)	Minor effect No scar
NN	Black tattoos	4 (Laser tried)	1.7 mm/ 0.4‐0.6‐1.2 J	3 (1‐5)	No effect No scar
BB	Black tattoos	7 (Untreated)	1.3 mm/ 0.9‐1.2 J	5 (4‐7)	Almost cleared, No scar (Minute site with skin thickening and another with thinning)
SØ	Red tattoos	3 (Laser tried)	1.3 mm/ 0.4 J	5 (4‐6)	Lost for follow‐up
MF	Red tattoos	3, 3 (Shaving and Laser tried)	1.3 mm/ 0.9 J	1 (0‐7)	Almost cleared, hypopigmentation No scar
MS	Black/colored tattoo	1 (Laser tried)	1.3 mm/ 0.9 J	6	Minor effect, 1 + scar
PH	Black/violet Tattoo	1 (Laser tried)	1.3 mm/ 0.9 J	3	Minor effect, No scar
TB	Black Tattoos	2 (Untreated)	1.7 mm/2.0 J 2.7 mm/2.0 J	5 5	Cleared. No scar Almost cleared. 1 + scar

### 3.1 Wheal and flare reactions immediately after HIFU – dose titration

**Figure 5:**
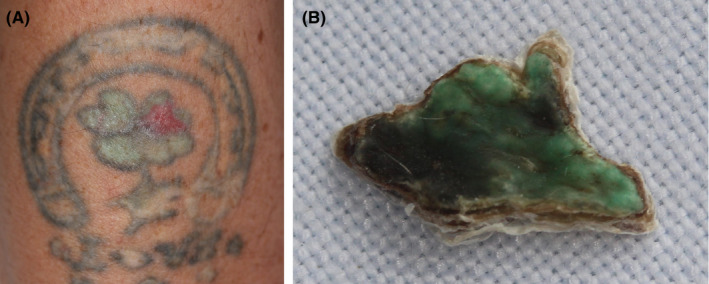
High‐intensity focused ultrasound (HIFU) dose titration test in a green tattoo (OW) previously treated with salabrasion and then by laser with rather poor outcome including remnant green pigment and scar formation. A, HIFU dose titration with three energy levels from 0.6 to 1.2 J/shot was made in different leaves of the flower motive. Green pigment was cleared in one area (1‐3 o'clock) treated with 1.2 J/shot, where significant wheal and flare were observed at treatment. Areas treated with lower doses had low wheal and flare reaction and showed no subsequent crustation. Remaining inflammation in treated spot after healing, which was ongoing at follow‐up. B, crust expelled from the wound during healing, turned downside up; green pigment of dermis is included in the crust and expelled when the crust loosens during the process of wound healing. The rather flat (inner) surface of the crust (upwards in figure) with bright green pigment representing the cleavage towards underlying dermis is noteworthy and indicates sharp delineation of the HIFU thermal insult towards underlying vital dermis

All subjects developed wheal and flare directly after treatment, most subjects grade 1 + and 2+, and a few with grade 3+. However, it was intended that 1 + and 2 + wheal and flare was used as an indicator of clinically relevant dose setting. In the first phase of the study, 12 subjects were assessed by dose titration, where 3‐4 doses of 0.4, 0.6, 0.9, and 1.2 J/shot were given to separate test sites in the tattoo or in the normal skin. The dose of best benefit/adverse effect ratio was chosen for further treatment and subsequently applied to the entire tattoo. The same dose was given in the next session (session number 2) after about 6 weeks when the course of healing had been observed. A green tattoo treated with a test dose titration is shown in Figure 5. The preferred dose also could be decided immediately from reading of the wheal and flare reaction in normal skin. *Pre‐treatment dose titration* was therefore deemed valuable.

### 3.2 Pain of treatment

All patients who had tried laser removal before HIFU rated painless by HIFU compared to lasers according to VAS (data not shown). Typical VAS pain scores of HIFU were between 5 and 6, compared to scores between 7 and 9 reported by the subjects who had experienced pain of laser treatment. Pain from HIFU was reported variable in the treated field with sporadic points of higher sensitivity as compared to HIFU treatment shots in other parts of the field. This was attributed to pain sensors that may be unevenly distributed over a skin surface. The HIFU‐related pain was described as being of short duration as compared to pain induced by lasers.

A single subject self‐administered an anesthetic topical (EMLA^®^, AstraZeneca) on the targeted tattoo prior to treatment. In this case, pain score was very low (VAS 0.5), while treatment response and post‐treatment healing remained within the range observed in the subjects.

### 3.3 Phases of wound, crustation, and healing of HIFU‐treated sites

Instantly on HIFU application, an epidermal reaction was obligatory, as intended. The epidermis became whitened, loose, and chapped. Occasionally, vesicles were seen.

Within a few days, a superficial crust formed, followed by denser and drier crustation. After 1‐2 weeks, a wound fully covered by a necrotic debris was observed. The necrotic tissue was extruded after 2‐8 weeks, in most cases leaving an open wound with a flat, excavated wound bed directly where HIFU had been dosed. In this phase, there was inflammation with redness and swelling of the wound margin involving some surrounding skin, the swelling increasing the impression of excavation. The wound gradually healed within 12 weeks, thus, relatively protracted. Two subjects had been treated by their practitioner with antibiotics, instituted in the phase of inflammation when infection is difficult to exclude. All subjects managed wound care themselves.

### 3.4 Efficacy of treatment

The tattoo was deemed cleared in 4 subjects (19%) and deemed partly cleared with some remnant pigment in 9 (43%) subjects (one subject lost for follow‐up). Thus, in total 13 subjects, that is, 62%, benefitted from the treatment with respect to full or partial clinically relevant tattoo pigment removal. Six subjects (28%) only had minor effect of HIFU. Two subjects (10%) had no effect, including the case with gun powder tattoo.

### 3.5 Side effects (scar)

Twelve (57%) had no scar, 5 (24%) had 1 + scar, and 4 (19%) had 2 + scar. Scar degree 3 + to 5 + was not observed. Effect versus scar was a scattered plot with no clear relationship. The two subjects with no effect had no scar and were considered non‐responders. One subject with hypopigmentation without a scar was registered.

## 4 CASE REPORT 1: BLACK TATTOO ACQUIRED TO SUPPORT RESEARCH OF FIRST APPLICATION OF 20 MHz HIFU TO TATTOO REMOVAL

**Figure 6:**
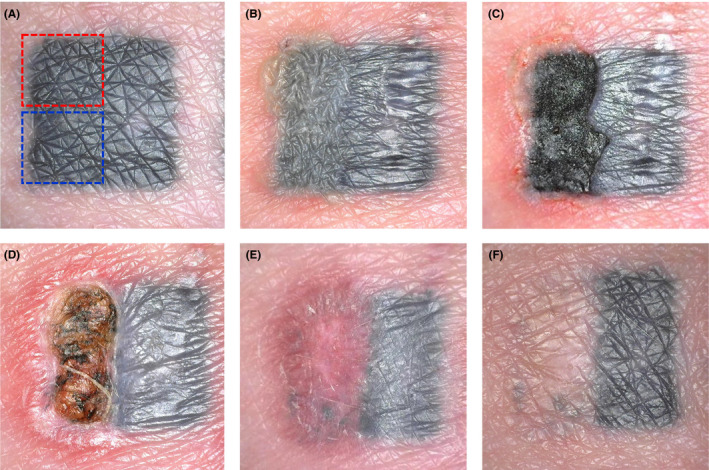
First treatment using 20 MHz high‐intensity focused ultrasound (HIFU) for tattoo removal, performed in a 48‐year‐old volunteer (TB), illustrating treatment effects and phases of healing. A, before HIFU treatment. The upper squared field marked red was treated with a probe with focus depth 1.7 mm, and the lower squared field marked blue was treated with a probe with focus depth 2.7 mm. Half of the tattoo served as untreated reference. B, immediately after HIFU treatments. Loosening and chapping of both treated fields are observed, with associated perilesional edema and disturbance of the untreated reference. Lacking edema in the treated fields contrasting untreated tattoo indicates damage to the vascular supply of the epidermis and outer dermis. C, 2 days post‐HIFU treatment. A confluent necrotic crust has formed. D, 10 days post‐HIFU treatment. The necrotic crust is expelled. The wound bed is flat and at a lower level in the field treated with the 2.7 mm probe (blue field) in comparison with the field treated with the 1.7 mm probe marked red. Reepithelization is active in the wound margin. There are major inflammation and swelling of the surrounding skin, with edema extending into the untreated reference site. E, 4 weeks post‐HIFU treatment. The epidermis is healed without restitution of skin markings, and the wound is closed. Ongoing inflammation in the dermis is causing redness. F, 20 weeks post‐HIFU treatment. Healing without scar is completed, with slight edema remaining. One‐session HIFU treatment has completely removed the tattoo, using the shallow probe (red field), while few discrete spots of pigment are remaining in the field treated with the deep probe (blue field). There is no sign of dyschromia, neither hypo‐ or hypermelanosis. Case supports to a high degree the hypothetic mode of action and course of healing introduced in Figure 2

Subject (TB) was a 48‐year‐old male volunteer, who had a squared black tattoo made on his right buttock for the purpose of testing. The tattoo was performed by a professional tattooist. The case is shown in Figure 6.

The upper left quarter of the tattoo (red test field) was treated in a single session with a probe with focal depth 1.7 mm targeting the outer dermis, and another quarter (blue test field) was treated with a probe focal dept 2.7 mm targeting the mid‐dermis to lower dermis level. Each quarter was given 25 shots with a duration of 200 ms/shot. The acoustic energy was approximately 4 J/shot. Due to the early and exploratory status of this treatment, higher settings than subsequently practiced were used.

The treatment response and healing phase followed the hypothetical model, shown in Figure 2, closely with initial loosening of the epidermis followed by a wound with crust and superficial necrosis, and with inflammation affecting the wound border, the surrounding skin and even the untreated reference area of the same tattoo; here, the skin surface became edematous with transient loss of surface markings. At end of observation, the black pigment of treated parts of the tattoo had gone, with the markings restored and no visual scar formation. There were a few spotty pigment remnants in the field treated with the 2.7 mm probe. A 1.3 mm probe was later in the study chosen as standard.

## 5 CASE REPORT 2: TATTOO REGRET, TEST TREATMENT OF A SELECTED BLACK TATTOO WHEN PICOSECOND LASER IN THE PAST HAD CAUSED SEVERE LOCAL REACTION, UNBEARABLE PAIN AND GENERAL MALAISE

Subject (SK) was a 33‐year‐old woman, Fitzpatrick Type 3, with ethnic predisposition to pigmentation. The subject had various tattoos with three on the arm causing social problems. Picosecond laser removal of a tattoo was attempted but given up, despite a new try with a low dose. The problem was unbearable pain and extraordinary swelling of the laser‐treated tattoo affecting her general condition and requiring analgesics and oral prednisone treatment.

A small black tattoo on the forearm, a 1.5 cm star, was selected for test treatment with HIFU, performed in one session, probe depth 1.1 mm, settings 150 ms, and 0.9 J/shot, with 20 shots applied. A pre‐test in normal skin with reading of wheal and flare had been performed.

**Figure 7:**
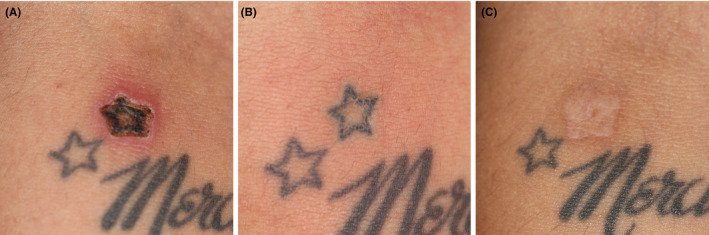
High‐intensity focused ultrasound (HIFU) tattoo removal (SK) of a small tattoo used to test the safety of 20 MHz HIFU (had experienced unbearable pain, swelling and malaise after picosecond laser). A, directly after HIFU treatment, very mild wheal and flare and just visible chapping of the treated tattoo (upper star with another star as reference). B, 10 days post‐HIFU; a necrotic crust has gone and an inflamed wound is seen, with edema and redness of the immediately surrounding skin. C, 7 months after treatment, 1 + scar with some skin markings restored, and discrete perilesional hypermelanosis following inflammation

Figure 7 illustrates the tattoo directly after treatment, after approximately 10 days and after 7 months. This tattoo was completely removed after one HIFU session leaving a mild scar rated 1+, however, with some upcoming skin markings. There was a narrow rim of postinflammatory hyperpigmentation in the surrounding skin, maybe with her ethnic predisposition as a background. It is estimated that the esthetic outcome will normalize over time. HIFU‐induced pain was rated 3 (1‐7), much less than experienced with the laser.

## 6 CASE REPORT 3: ALLERGY TO RED TATTOO PIGMENT, INCOMPLETE REMOVAL BY DERMATOME SHAVING, REMOVAL OF REMNANT PIGMENT WITH HIFU

Subject (MJ) was a 29‐year‐old man with many tattoos. A tattoo on his upper arm sized 3 × 3 cm developed allergy to red pigment, manifested in the entire tattoo as a “plaque elevation” type of allergy with major inflammation, thickening, and itch. Dermatome shaving was performed and had released most of the pigment and the symptoms, but three spots of remnant pigment and a rim of weaker pigment at the margin of the tattoo remained. Dermatome shaving had caused grade 2 + scar.

High‐intensity focused ultrasound was given in one session with approximately 50 shots and a 1.1 mm focal depth probe, settings 0.9 J/shot, and pulse duration 150 ms.

**Figure 8:**
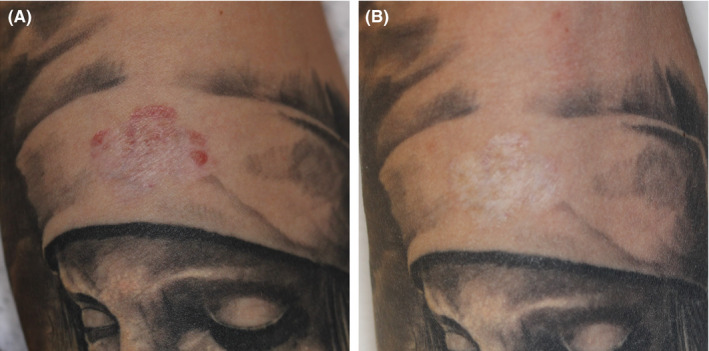
High‐intensity focused ultrasound (HIFU) as back‐up removal of remnant red pigment in a subject (MJ) with tattoo allergy, previously treated with dermatome shaving. A, before HIFU treatment. B, 10 months post‐HIFU; removal was completed and the scar and the esthetic outcome acceptable. Subject was ready for a cover‐up tattoo with a non‐red color. The allergen had been cleared

Figure 8 illustrates the red pigmentation before HIFU treatment and at a follow‐up visit after 10 months. The remnant red pigmentation has been completely removed, and subject had not experienced any allergic reaction during the healing phase.

## 7 CASE REPORT 4: TATTOO CAUSING SOCIAL INCOMPETENCE, WITH A SPECIAL NEED OF FAST REMOVAL

**Figure 9:**
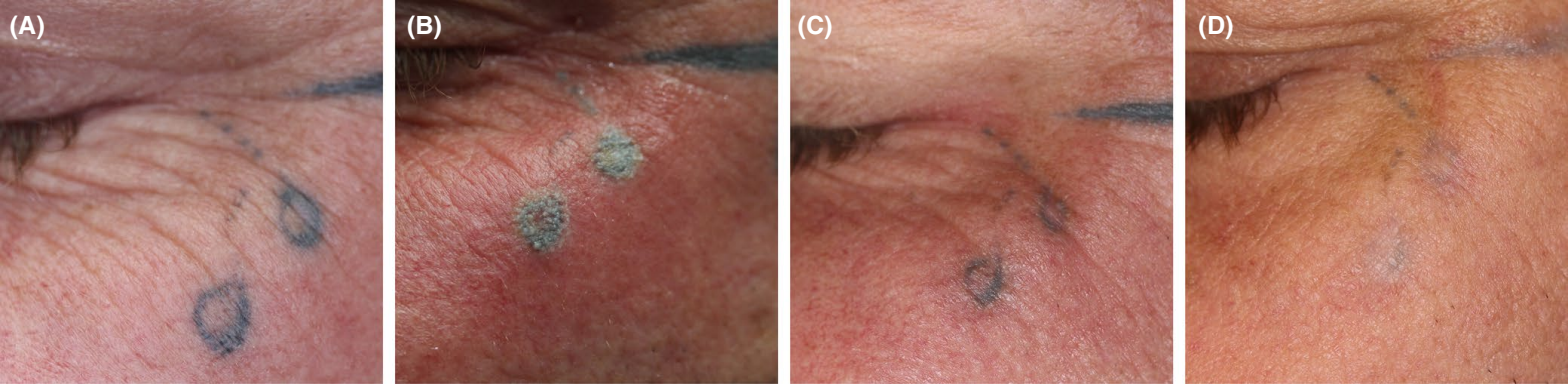
Droplets under eye as a marker of serious event or danger, of high social impact. A, before high‐intensity focused ultrasound (HIFU). B, directly after the first HIFU treatment; chapping of treated field after ultrasound shots, and wheal and flare reaction. C, status after first treatment after healing. D, a second treatment with HIFU was given; removal is completed with no significant remnant pigment and no scar. The need for swift treatment was high priority and had been met. The tattoos were made with a liner needle; thus, the pigment was installed deeply in the skin

Subject was a younger man, who carried two tattooed droplets under the eye, with strong negative social impact and risks. He also had various larger tattoos on the head. Previous laser treatments had not reduced the color significantly. He was treated with HIFU, 1.1 mm probe and 0.9 J/shot, but the effect was barely visible. A second treatment was given with a 1.3 mm probe and 1.2 J/shot. A satisfactory result with no sequelae was noted after 4 months (Figure 9).

## 8 DISCUSSION

In this introduction of 20 MHz HIFU for tattoo removal, the effectiveness of HIFU was confirmed with 62% benefitting from HIFU. Nevertheless, many subjects were only partly cleared from pigment or had minor effect, and only a few were completely cleared.

However, HIFU was put on a real acid test, where no method was likely to produce ideal results. HIFU was used as a rescue when Nd:YAG, picosecond lasers, and dermatome shaving had failed or caused adverse effects or sequelae. Seen in this perspective, HIFU provided therapeutic results in cases where surgical excision could be a final solution. Excision is however only possible in some locations and in small tattoos and furthermore carries a risk of disfiguring contraction disturbing the normal skin surface contour. CO_2_ lasers are controversial as last‐option tattoo removal and have a high risk of producing scars, which can be severe, particularly if the treatment is applied on the top of preexisting scarring of the treated lesion due to previous treatment failures.

Nd:YAG and picosecond lasers remain first‐line methods for tattoo removal in simple tattoo regret and have many advantages. Lasers are nevertheless hampered by imperfections: color dependency, high pain level, requires many treatments, takes a long time (it takes a year or so to complete a full treatment schedule), expensive, confined with risk of scar and dyspigmentation as sequelae. Last, but not least, the modest efficiency in clearing of pigment has given documented suboptimal patient satisfaction.^8^

Laser removal of tattoos is a big industry, but the method is primarily established through practical experience of treatment providers. Widespread use contrasts the surprisingly limited documentation of efficacy, side effects, and customer satisfaction in the medical literature.^3^, ^4^, ^8^

The presumptive pros and cons of lasers and HIFU are presented in Table 2. Comparison of lasers versus HIFU has the following highlights: Lasers are hampered by the limitation of colors that can be treated, the need of many sessions and long treatment schedules, expensive equipment and high price for the customer, who is prone to become non‐adherent and stop the treatment course too early. HIFU is hampered by being ablative thus more aggressive with a longer wound healing phase and potential risk of creating scar, albeit the depth control is optimized and not variable, free‐hand and very operator‐dependent as ablation with CO_2_ laser is.

**Table 2:** Presumptive pros and cons of tattoo removal by nanosecond Q‐switched Nd:YAG lasers and 20 MHz high‐intensity focused ultrasound (HIFU). With recent picosecond lasers, the number of sessions to complete a full treatment course is fewer and may be about 6

	Nd:YAG laser	20 MHz HIFU
Color dependency	Yes	No
Completed treatment course, number of sessions	8‐12	1‐3
Length of full treatment, months	12‐18	1‐3
Efficiency pigment removal, rated from 0 to 3	2‐3/variable	2/variable^a^
Can be used in larger coherent tattoos	Yes	No
Can be used to treat allergic tattoo reactions (red azo pigment)	No	Yes
Pain on treatment, rated from 1 to 3	3	1‐2
Wound healing time post‐treatment, months	<1	1‐3
Risk of scar post‐treatment, rated from 1 to 3	0‐2	1‐3
Photochemistry, hazardous chemicals/allergens from pigment	Yes	No

^a^ Needs further study. HIFU primarily applied to difficult tattoos, when lasers and other methods of removal had failed.

The ablative nature of HIFU might however offer the advantage of shorter and more realistic treatment schedule, typically 1‐2 sessions. This can be particular important for subjects, who have urgent need for removal, for example, ex‐gang members in the process of resocialization or subjects who seek career paths, where tattoos are either a disqualifying feature or connected to general negative stigmatization. There is furthermore a large group of tattooed, who require swift removal to prepare a new or cover‐up tattoo. The largest field of application is however cases where lasers fail to do the job in a reasonable time, and cases where lasers are too painful or cause major problems. A special potential indication of HIFU is for tattooed persons with allergy to red pigment. Lasers are risky in these patients, since allergenic breakdown products of the red azo pigment may be produced though the photochemical activity of the laser on the pigment, causing allergy burst and even anaphylactic crisis.

High‐intensity focused ultrasound has the significant disadvantage that crustation and wound healing take longer time than healing after lasers. This may however be seen as beneficial for removal of the pigment; pigment from dermis deep remain being expelled via the wound, and inflammation in the wound may facilitate biochemical breakdown and digestion of remnant pigments under and around the treated tattoo.

The future use and position of 20 MHz tattoo removal seem primarily to be as first‐line treatment of smaller black or multicolored tattoos in persons, who need the treatment to be swift. Another first‐ or second‐line potential indication is difficult tattoos, where the outcome of laser removal is a failure or suboptimal due to poor match of the wavelength of the laser source and the absorbance of the pigment. There is presently no experience with HIFU applied to large tattoos, and the size of the tattoo is a logic limitation of HIFU because of the longer healing phase of the ablation wound.

Twenty megahertz HIFU was recently documented effective in the treatment of actinic keratosis, with a cure rate of 97%.^28^ HIFU was found to be well‐suited for field eradication and has the potential to replace or supplement photodynamic therapy. HIFU was also applied to skin cancers. HIFU is being studied for other indications: venous spiders, venous lakes, hemangiomas, lentigines, and benign skin tumors, thus common concerns in the elderly population.

In conclusion, 20 MHz HIFU has a significant potential for future use in dermatological clinics, particularly in the laser clinics as a new and specialized member of the family of advanced machines. There is also a hitherto little studied potential in the beauty industry.

High‐intensity focused ultrasound is an advanced piece of equipment, and skills and experience of the operator are certainly needed as it is the case with lasers. Thus, this new method shall be met respectfully, and first‐time users shall invest proper time for study, learning, and practical training before clients or patients are treated.

## CONFLICT OF INTEREST

This study has been funded by TOOsonix A/S, Denmark.

## References

[1] Serup J , Kluger N , Bäumler W (eds.). Tattooed skin and health. Curr Probl Dermatol. 2015;48:1‐258.25833618

[2] Serup J , Bäumler W (eds.). Diagnosis and therapy of tattoo complications, with atlas of illustrative cases. Curr Probl Dermatol. 2017;52:1‐234. 28288465 10.1159/000453379

[3] Bäumler W . Laser treatment of tattoos: basic principles. Curr Probl Dermatol. 2017;52:94‐104. 28288450 10.1159/000450809

[4] Karsai S . Removal of tattoos by Q‐switched nanosecond lasers. Curr Probl Dermatol. 2017;52:105‐112. 28288448 10.1159/000450811

[5] Adatto MA , Amir R , Bhawalkar J , et al. New and advanced picosecond lasers for tattoo removal. Curr Probl Dermatol. 2017;52:113‐123. 28288459 10.1159/000450812

[6] Engel E , Spannberger A , Vasold R , König B , Landthaler M . Photochemical cleavage of a tattoo pigment by UVB radiation or natural sunlight. J Dtsch Dermatol Ges. 2007;5:583‐589. 17610608 10.1111/j.1610-0387.2007.06333.x

[7] Engel E , Vasold R , Santarelli F , et al. Tattooing of skin results in transportation and light‐induced decomposition of tattoo pigments—a first quantification in vivo using a mouse model. Exp Dermatol. 2009;19:54‐60. 19703227 10.1111/j.1600-0625.2009.00925.x

[8] Hutton Carlsen K , Esmann J , Serup J . Tattoo removal by Q‐switched YAG laser: client satisfaction. J Eur Acad Dermatol Venereol. 2017;31:904‐909. 28107564 10.1111/jdv.14124

[9] Serup J , Bäumler W . Guide to treatment of tattoo complications and tattoo removal. Curr Probl Dermatol. 2017;52:132‐138. 28288463 10.1159/000452966

[10] Sepehri M , Jørgensen B , Serup J . Introduction of dermatome shaving as first line treatment of chronic tattoo reactions. J Dermatol Treat. 2015;26:451‐455. 10.3109/09546634.2014.999021 25672517

[11] Hutton Carlsen K , Serup J . Sequels to tattoo removal by caustic products. Skin Res Technol. 2018;24:636‐641. 29782050 10.1111/srt.12578

[12] Wollina U . Depigmentation and hypertrophic scars after application of a fluid lactic acid tattoo eraser. Wien Med Wochenschr. 2015;165:195‐198. 25962974 10.1007/s10354-015-0353-x

[13] Focused Ultrasound Foundation . State of the Field 2019. https://www.fusfoundation.org/. Accessed Match 20, 2020.

[14] Ellens NPK , Partanen A . Preclinical MRI‐guided focused ultrasound: a review of systems and current practices. IEEE Trans Ultrasonics, Ferroelectr Freq Control. 2017;64(1):291‐305. 10.1109/TUFFC.2016.2609238 27662675

[15] Stephanie G , Peters M , Arya M , et al. A multicentre study of 5‐year outcomes following focal therapy in treating clinically significant nonmetastatic prostate cancer. Eur Urol. 2018;74:422‐429. 29960750 10.1016/j.eururo.2018.06.006 PMC6156573

[16] Quinn SD , Gedroyc WM . Thermal ablative treatment of uterine fibroids. Int Jour Hyperthermia. 2015;31(3):272‐279. 25815582 10.3109/02656736.2015.1010608

[17] Barile A , Arrigoni F , Zugaro L , et al. Minimally invasive treatments of painful bone lesions: state of the art. Med Oncol. 2017;34(53):1‐11. 28236103 10.1007/s12032-017-0909-2

[18] Kim M , Jung NY , Park CK , et al. Comparative evaluation of magnetic resonance‐guided focused ultrasound surgery for essential tremor. Stereotact Funct Neurosurg. 2017;95:279‐286. 28810261 10.1159/000478866

[19] Lamsam L , Johnson E , Connolly ID , Wintermark M , Hayden Gephart M . A review of potential applications of MR‐guided focused ultrasound for targeting brain tumor therapy. Neurosurg Focus. 2018;44(2):E10. 10.3171/2017.11.FOCUS17620 29385922

[20] Peek MCL , Wu F . High‐intensity focused ultrasound in the treatment of breast tumours. Ecancer. 2018;12(794):1‐10. 10.3332/ecancer.2018.794 PMC5804717 29434660

[21] Lang BH , Wu ALH . High intensity focused ultrasound (HIFU) ablation of benign thyroid nodules—a systematic review. J Therapeutic Ultrasound. 2017;5(11):1‐9. 10.1186/s40349-017-0091-1 PMC5434558 28523127

[22] Zawada T , Bove T . Acoustic device for skin treatment and methods of using the same. Patent application WO. 2018/158355 A1.

[23] Bove T , Zawada T , Serup J , Jessen A , Poli M . High‐frequency (20‐MHz) high‐intensity focused ultrasound (HIFU) system for dermal intervention: preclinical evaluation in skin equivalents. Skin Res Technol. 2019;25:382‐388. 30620418 10.1111/srt.12661

[24] Saaki GH , Tevez A . Clinical safety and focused‐image ultrasonography: a 2‐year experience. Aesthet Surg J. 2012;32(5):601‐612. 22531061 10.1177/1090820X12445576

[25] Day D . Microfocused ultrasound for facial rejuvenation: current perspectives. Res Rep Focus Ultrasound. 2014;2:13‐17.

[26] TOOsonix System ONE‐R system description. Available from https://www.toosonix.com/. Accessed March 20, 2020.

[27] Soegaard S , Aarup V , Serup J , Bove T , Zawada T , Jessen A , Poli M‐F . (20 MHz) high‐intensity focused ultrasound system for dermal intervention: a 12‐week local tolerance study in minipigs. Skin Res Technol. 2020;26:241‐254. 31541524 10.1111/srt.12786

[28] Serup J , Bove T , Zawada T , et al. High frequency (20 MHz) high‐intensity focused ultrasound (HIFU): treatment of actinic keratosis, basal cell carcinoma and Kaposi sarcoma. Skin Res Technol. 2020. 10.1111/srt.12883 PMC7754281 32557832

[29] Serup J , Sepehri M , Hutton CK . Classification of tattoo complications in a hospital material of 493 adverse events. Dermatology. 2016;232:668‐678. 27974717 10.1159/000452148

[30] Sepehri M , Hutton Carlsen K , Serup J . Papulo‐Nodular reactions in black tattoos as markers of sarcoidosis: study of 92 tattoo reactions from a hospital material. Dermatology. 2016;232:679‐686. 28166524 10.1159/000453315

[31] Serup J , Hutton Carlsen K , Dommershausen N , et al. Identification of pigments related to allergic tattoo reactions in 104 human skin biopsies. Contact Dermatitis. 2020;82:73‐82. 31626330 10.1111/cod.13423 PMC6973263

[32] Hutton Carlsen K , Sepehri M , Serup J . Tattooist‐associated tattoo complications: “Overworked Tattoo,” “Pigment Overload” and infections producing early and late adverse events. Dermatology. 2020;236(3):208‐215. 31499492 10.1159/000501962

